# Fatal Overdose with the Cannabinoid Receptor Agonists MDMB-4en-PINACA and 4F-ABUTINACA: A Case Report and Review of the Literature

**DOI:** 10.3390/toxics11080673

**Published:** 2023-08-05

**Authors:** Gábor Simon, Mónika Kuzma, Mátyás Mayer, Karola Petrus, Dénes Tóth

**Affiliations:** 1 Department of Forensic Medicine, Medical School, University of Pécs, Szigeti str. 12, H-7624 Pécs, Hungary; monika.kuzma@aok.pte.hu (M.K.); matyas.mayer@aok.pte.hu (M.M.); karola.petrus@aok.pte.hu (K.P.); denes.toth@aok.pte.hu (D.T.); 2 Department of Laboratory Medicine, Medical School, University of Pécs, Szigeti str. 12, H-7624 Pécs, Hungary

**Keywords:** forensic pathology, forensic toxicology, synthetic cannabinoid, MDMB-4en-PINACA, 4F-ABUTINACA, overdose, autopsy

## Abstract

A case of a 26-year-old male who died from consuming synthetic cannabinoid receptor agonists MDMB-4en-PINACA and 4F-ABUTINACA is reported. MDMB-4en-PINACA and 4F-ABUTINACA are potent synthetic cannabinoid receptor agonists (SCRAs). This is the first detailed reporting of MDMB-4-en-PINACA and 4F-ABUTINACA associated fatality, which can help the routine forensic work. The scientific literature on the symptoms associated with these substances are evaluated, along with the pharmacological properties and possible mechanism of death. A forensic autopsy was performed according to Recommendation No. R (99)3 of the Council of Europe on medico-legal autopsies. Histological samples were stained with hematoxylin and eosin (HE). Complement component C9 immunohistochemistry was applied to all heart samples. Toxicological analyses were carried out by supercritical fluid chromatography coupled with tandem mass spectrometry (SFC-MS/MS) and headspace gas chromatography with a flame ionization detector (HS-GC-FID). The literature was reviewed to identify reported cases of MDMB-4en-PINACA and 4F-ABUTINACA use. Autopsy findings included brain edema, internal congestion, petechial bleeding, pleural ecchymoses, and blood fluidity. Toxicological analyses determined 7.2 ng/mL of MDMB-4en-PINACA and 9.1 ng/mL of 4F-ABUTINACA in the peripheral blood. MDMB-4en-PINACA and 4F-ABUTINACA are strong, potentially lethal SCRA, and their exact effects and outcome are unpredictable.

## 1. Introduction

Synthetic cannabinoid receptor agonists (SCRAs) first appeared as a legal alternative to cannabis in 2004, and they have been linked to numerous fatalities since then [1]. SCRAs have diverse toxicological effects, including renal injury (proximal tubular dilation and tubular necrosis), cardiotoxicity (arrhythmias, myocardial infarction, heart failure, cardiomyopathies), respiratory depression, gastrointestinal symptoms (abdominal pain, vomiting), epilepsy, and a broad spectrum of psychiatric symptoms [2,3,4,5,6]. SCRA toxicity can be related to fatalities directly (alone or mono intoxication), or indirectly (behavioral and physical contribution), but many times their contributory role is not clear [7,8]. The main problem with the forensic evaluation of potentially SCRA-related fatalities is that the concentration threshold that could be fatal is poorly defined and exhibits significant variations, especially in light of the several different SCRAs consumed [8]. Detailed reports—including autopsy findings and toxicological results—of fatal cases associated exclusively or partially with SCRAs (and other psychoactive substances) can be advised to help understand their mechanism of effect and establish potentially toxic and fatal concentrations for each substance, thus helping everyday forensic practice. It is especially important in the case of widely used compounds.

MDMB-4en-PINACA—also known as MDMB-PENINACA, 5Cl-ADB-A, or ADB-PINACA-A, with the IUPAC name methyl (S)-3,3-dimethyl-2-(1-(pent-4-en-1-yl)-1H-indazole-3-carboxamido)butanoate—is a full and potent SCRA. It was first identified in Europe in 2017 [9], appeared in the market in mid-2019, and became one of the most common SCRA in the next year and a half [10,11,12]. It quickly became one of the prisons’ most commonly detected SCRA [13,14]. Additionally, there are reports that cannabis is regularly adulterated with MDMB-4en-PINACA [14,15]. MDMB-4en-PINACA concentrations in these adulterated cannabis flower samples ranged from 0.3 to 4.6 µg/mg, whereas in hashish samples, it varied from 1.7 to 7.2 µg/mg [15].

**Figure 1:**
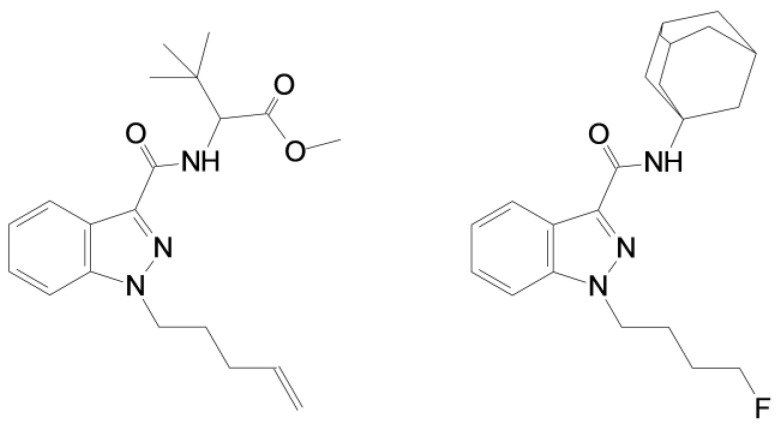
Chemical structure of MDMB-4en-PINACA (**left**) and 4F-ABUTINACA (**right**).

MDMB-4en-PINACA shares structural features with several SCRAs, including 4F-MDMB-BINACA and 5F-MDMB-PINACA. Similarly to these compounds, MDMB-4en-PINACA contains an indazole core, a carboxamide link, and a tert-leucinate (dimethyl methyl butanoate) group. However, it differs in the tail from these compounds; it has a pent-4-ene moiety on a pentyl tail (Figure 1).

4F-ABUTINACA—also known as N-(4-fluorobutyl)-APINACA or 4F-ABINACA, with the IUPAC name N-(1-adamantyl)-1-(4-fluorobutyl)indazole-3-carboxamide—is a fourth generation indazole-adamantyl-derived SCRA (Figure 1). Its chemical structure is most similar to APINACA (AKB48) and 5F-APINACA (5F-AKB48), which are potent agonists of both the CB1 receptor and the CB2 receptor [16]. 4F-ABUTINACA appeared in Asia in 2020, but no data are available about its pharmacological properties [17,18,19].

Based on in vitro biological activity assessment at cannabinoid type 1 (CB1) receptor via its interaction with β-arrestin 2, the potency and efficacy of MDMB-4en-PINACA is similar to that of their structural analogs 4F-MDMB-BINACA and 5F-MDMB-PINACA. In vitro studies showed an EC50 of 1.88–2.47 nM, an Emax of 221–299% (compared to JWH-018), and a Ki value of 0.28 on the CB1 receptor [20,21,22,23]. MDMB-4en-PINACA seems to have a seven-fold greater affinity to cannabinoid type 2 (CB2) receptor than CB1 receptor [24].

Since MDMB-4en-PINACA has not been formally studied in humans, information about its pharmacological properties is limited [1]. An in vivo study revealed that MDMB-4en-PINACA interferes with hippocampal functions and impairs cognitive performance, highlighting the cognitive harm posed by MDMB-4en-PINACA [25].

It metabolizes rapidly [26], with an in vitro half-life (t1/2) of 9.1 min [1]. Gu et coworkers found that MDMB-4en-PINACA metabolism involves 11 metabolic pathways, including acetylation, a novel metabolic pathway for SCRAs. According to their findings, the major metabolic pathways involved in MDMB-4en-PINACA metabolism are ester hydrolysis and hydroxylation, and up to nine metabolites can be detected in the serum [27]. Xiang et al. also identified further ketone metabolites and special phase II metabolites [28]. Various clinical symptoms were reported after using MDMB-4en-PINACA, such as headaches, seizures, paranoia, anxiety, hallucinations, amnesia, mydriasis, nausea, and vomiting [29,30].

The European Monitoring Centre reported four deaths with confirmed exposure to MDMB-4en-PINACA for Drugs and Drug Addiction (EMCDDA). These cases occurred between 2019 and 2020, but the details were not published [31].

At the time of writing, this is the first fatal case report involving 4F-ABUTINACA reported with detailed autopsy findings and concentrations.

## 2. Case Report

The deceased was a 26-year-old Caucasian male with no prior medical history. He had a long history of abusing new psychoactive substances (NPS) and had previously committed multiple drug-related offenses. He was wearing an electronic tagging device at the time of his death. He had complained of acute headache and angina the day before his death. On the morning of his death, he went to the restroom at approximately 5:45 a.m. He returned to his room a few minutes later. In a short while, his father heard a thump and entered his room. The victim had a weak pulse and noisy breathing (death rattle). The father immediately called an ambulance and began resuscitation. The emergency medical personnel noticed pulseless electrical activity. Resuscitation efforts were unsuccessful, and he was pronounced dead at the scene. The police found 6.82 g of dried, shredded, green plant material in a mini zip log bag and a few cigarette butts.

Analysis of the seized green plant material and cigarette butt was carried out by the exclusively authorized national laboratory (Hungarian Institute of Forensic Sciences), MDMB-4en-PINACA and 4F-ABUTINACA were detected in each sample. The authors received the results of this analysis 24 months after the autopsy.

## 3. Materials and Methods

### 3.1. Autopsy and Sampling

Forensic autopsy was performed according to the Recommendation No.R (99)3 of the Council of Europe [32] on medico-legal autopsies. Histological samples were collected from the brain (posterior limb of the internal capsule with adjacent thalamus, rostral pons, cerebellum including the dentate nucleus, hippocampus, dorsal frontal inter-arterial watershed zone), lungs, heart (sinoatrial node, Koch’s triangle (AV node), right ventricle, septum, and left ventricle), suprarenal glands, kidneys, and liver. Toxicological samples were collected from the femoral vein (whole blood) and bladder (urine).

### 3.2. Histology

All samples were fixed with 9% buffered formalin for three days, then stained with hematoxylin and eosin (HE). Immunohistological staining for complement factor C9 (cat. no. ABS 004-22-02, Thermo Fisher Scientific, Rockford, IL, USA) was applied on all heart samples.

### 3.3. Toxicological Analyses

Toxicological analyses were carried out by supercritical fluid chromatography tandem mass spectrometry (SFC-MS/MS) (Waters^®^ ACQUITY UPC2 supercritical fluid chromatograph coupled with Xevo TQ-S triple quadrupole mass spectrometer, Milford, MA, USA), and headspace gas chromatography with flame ionization detector (HS-GC-FID) (Agilent Technologies G1888 headspace with 7890A gas chromatograph system, Santa Clara, CA, USA). Prior to the analyses, biological samples (blood and urine) were stored at 4 °C. After the analytical measurements, the samples were stored frozen (−20 °C).

SFC-MS/MS was used to identify and quantify 295 compounds, including drugs (e.g., antihypertensive drugs, anxiolytics, antipsychotics, antidepressants, antiepileptics, general and local anesthetics, NSAIDs, opioids, anticoagulants), narcotics, and novel psychoactive substances (e.g., SCRAs). In the first step of sample preparation, nine isotope-labeled internal standards were added to the samples, which cover a wide range of physicochemical properties to monitor the extraction procedure for all the target molecules. The isotope-labeled standards were amphetamine-D6, 4-methylmethcathinone-D3, delta-9-tetrahydrocannabinol-D3, 11-nor-9-carboxy-tetrahydrocannabinol-D3, N-[(1S)-1-(aminocarbonyl)-2-methylpropyl]-1-[(4-fluorophenyl)methyl]-1H-indazole-3-carboxamide D4 (AB-FUBINACA-D4), carbamazepine-D10, citalopram-D6, alprazolam-D5, and clonazepam-D4, all were purchased from Lipomed AG (Arlesheim, Switzerland). The internal standard of SCRAs was AB-FUBINACA-D4. The SCRA reference standards were obtained from the exclusively authorized national laboratory (Hungarian Institute for Forensic Sciences, Budapest, Hungary). Metabolites of SCRAs are not analyzed routinely in our laboratory.

The HS-GC-FID method was applied to determine alcohols (methanol, ethanol, 1-propanol, 2-propanol, n-butanol) and other volatiles (e.g., acetone, toluene, ethyl acetate). Tert-butanol was used as an internal standard.

The applied analytical procedures were evaluated for a number of validation characteristics (selectivity, repeatability and intermediate precision, limit of detection, limit of quantification, and calibration range).

#### 3.3.1. SFC-MS/MS Conditions

Measurements were performed by an ACQUITY UPC2 supercritical fluid chromatography system (Waters) coupled with a Xevo TQ-S Triple Quadrupole Mass Spectrometer (Waters). Data were recorded by MassLynx software.

Separation of compounds was performed at 45 °C on a 2.1 mm × 100 mm, 1.7 μm particle size, Waters ACQUITY Torus^TM^ DIOL analytical column with a guard cartridge (Torus^TM^ DIOL VanGuard^TM^, 2.1 mm × 5 mm, 130 Å, 1.7 μm). The mobile phase consisted of a mixture of carbon dioxide (A) and 10 mM ammonium hydroxide, and 12 mM formic acid in methanol/water (97.2/2.8, *v*/*v*) (B). The following gradient profile was used: 97.5% A at 0 min and 37.5% A at 10 min. A pre-equilibration period lasting 2.5 min was applied before each injection. The flow rate of the mobile phase was 0.6 mL/min, the injected volume was 0.5 μL. Constant 175 bar back pressure was used to maintain the supercritical state. To sustain a suitable electrospray, methanol was added to the mobile phase as a makeup solvent with a flow rate of 60 μL/min (Waters 515 HPLC Pump). The MS measurement was performed in positive ion mode (except for some acidic compounds such as barbiturates). The ESI source was operated with a spray voltage of 3 kV in both positive and negative ion modes. Cone voltage was 30 V. The source was set at 150 °C. Both desolvation and cone gases were nitrogen delivered at 300 and 150 L/h, respectively. Desolvation gas was tempered at 300 °C. The collision gas was argon with a flow rate of 0.13 mL/min. MS/MS experiments were performed in MRM (multiple reaction monitoring) mode with an isolation window of 0.4 *m*/*z*. MRM transitions of MDMB-4en-PINACA and 4F-ABUTINACA can be seen in Table 1.

Peak detection and quantification were achieved using TargetLynx XS software (Waters). The observed ions (mass in *m*/*z*) were accepted and quantified if the following conditions were met: appropriate MS1 mass, appropriate retention time, appropriate MS2 mass, appropriate fragmentation pattern (three MRM transitions with appropriate peak area ratios), and recovery of internal standard.

#### 3.3.2. Sample Preparation—SALLE (Salting out Assisted Liquid-Liquid Extraction)

One hundred and twenty μL of internal standard solution (125 mM formic acid/acetonitrile) was added to 90 μL of the sample. After vortex-mixing, the mixture was allowed to stand at room temperature for 5 min. In the next step, exact amount of solid ammonium formate as salting agent was added to the mixture to obtain a saturated solution at 20 °C and incubated in a thermomixer (20 °C, 1200 rpm) for 15 min. After the incubation mixture was centrifuged (18,000 rcf, 20 °C) for 5 min, 30 μL of the supernatant was transferred into a microinsert from which 0.5 μL was injected to the chromatographic system.

## 4. Review of the Literature

The literature was reviewed to identify reported cases of MDMB-4en-PINACA and 4F-ABUTINACA use. The search was performed on 25 July 2023 using electronic databases of PubMed, Scopus, and Web of Science (WoS) using the following search parameters: MDMB-4en-PINACA (all fields) and 4F-ABUTINACA (all fields). Conference abstracts and works in languages other than English were excluded. Results from all three databases were downloaded and collated for duplicates. Manuscripts not describing a case or cases without information on the concentration, symptoms, or autopsy findings were excluded.

## 5. Results

### 5.1. Autopsy

The autopsy was performed five days after death and documented a well-developed, athletic build man without signs of significant trauma. A puncture wound over the lateral aspect of the left cubital fossa and small abrasions on the chest were found as a result of resuscitation. Neither petechiae nor rash were identified. Internal examination revealed brain edema (1670 g), internal congestion, petechial bleeding, pleural ecchymoses, and fluidity of the blood. No anatomic cause of death was found. The results of the histopathological examination were unremarkable except for mild subendocardial fibrosis. The main macro- and microscopic findings are summarized in Table 2.

### 5.2. Toxicology

Toxicological analyses determined 7.2 ng/mL of MDMB-4en-PINACA in the peripheral blood and 0.4 ng/mL in the urine. Other substances identified in the blood were caffeine (126 ng/mL) and theophylline (26 ng/mL). Ethyl alcohol could not be detected.

**Figure 2:**
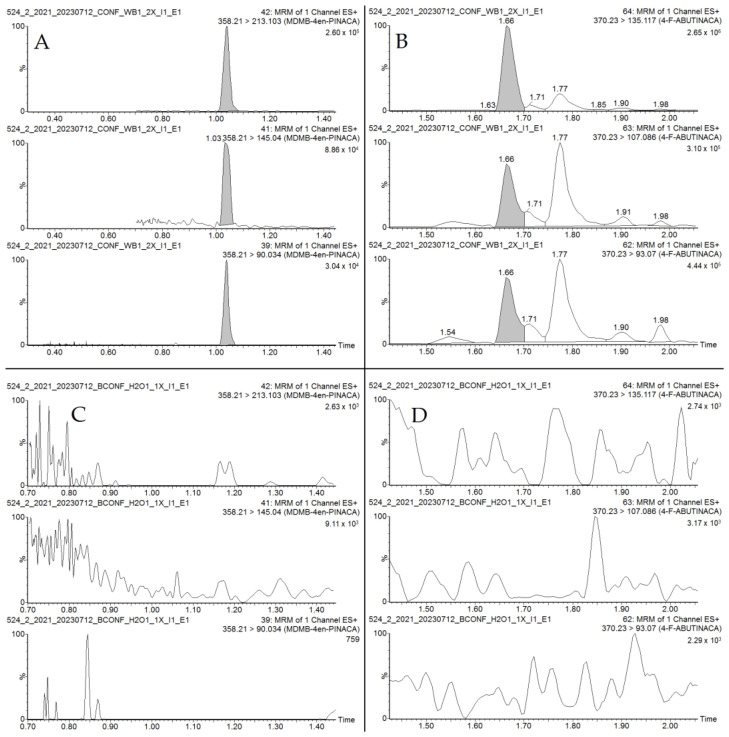
Representative MRM chromatograms of MDMB-4en-PINACA (**A**) and 4F-ABUTINACA (**B**) in postmortem peripheral blood sample, and corresponding chromatograms of a blank sample (**C**,**D**).

Blood and urine samples were re-examined 24 months after the autopsy, because analysis of the seized green plant material and cigarette butt revealed that these contained 4F-ABUTINACA in addition to MDMB-4en-PINACA. At the time of the autopsy, 4F-ABUTINACA was not routinely examined in our laboratory. The repeated toxicological analyses determined 9.1 ng/mL of 4F-ABUTINACA in the peripheral blood and 2.0 ng/mL in the urine (Figure 2). Furthermore, it turned out that MDMB-4en-PINACA is stable during long-term storage (24 months) at −20 °C both in the blood and the urine. Moreover, 103.8% of the previously determined concentration was present in unchanged form in the blood, and 100.6% in the urine. Since the stability test of 4F-ABUTINACA could not be performed, the possibility that its concentration was much higher at the time of the autopsy cannot be excluded [33].

The limit of detection (LOD) of the applied method was 0.09 ng/mL for MDMB-4en-PINACA and 4F-ABUTINACA. The limit of quantification (LOQ) was 0.15 ng/mL for MDMB-4en-PINACA and 4F-ABUTINACA. Calibration range was 0.15–50.0 ng/mL for both compounds.

### 5.3. Review of the Literature

MDMB-4-en-PINACA was first mentioned in the scientific literature in 2019 [34]. There are now (July 2023) 44 scientific articles in all (40 entries in Pubmed, 41 in Scopus, and 37 in Web of Science), but none of these describe a fatal case in detail, and only one describes the symptoms linked with its usage. Goncalves et al. reported eight cases of MDMB-4en-PINACA associated hospitalization with the most common symptoms of paranoia and/or hallucinations (four cases), nausea and/or vomiting (three cases), altered or loss of consciousness (three cases), psychomotor agitation and/or aggressiveness (three cases), headaches (three cases), persistent tiredness (three cases), mydriasis (three cases), seizure (two cases), amnesia (two cases), and dizziness (two cases) [29]. Seizures after consumption of MDMB-4-en-PINACA were also reported [30]. There was only one paper in which a case of a 40-year-old female was reported who died because of mixed drug toxicity, including MDMB-4-en-PINACA [35]. However, there is no information on its concentrations or any postmortem findings. Ricciardo reported a case of finding MDMB-4en-PINACA in a case of fatal thyroid storm, but they were not able to quantify the MDMB-4-en-PINACA due to prior embalming [36].

Regarding the 4F-ABUTINACA, the search revealed only one scientific article describing a UPLC method for the simultaneous determination of five indole/indazole amide-based SCRAs including 4F-ABUTINACA (article is in Chinese) [37], and it is mentioned only in one other article [38].

## 6. Discussion

Cardiac arrhythmias and myocardial infarction are the most common causes of death in SCRA-related fatalities, although other direct (respiratory depression, excited delirium) or indirect (accident, suicide) mechanisms are also frequent [38,39,40,41]. In fatal incidents, the most common signs and symptoms are sudden collapse, chest pain, and seizures [7]. Autopsy findings of SCRA-related fatalities are diverse. Internal congestion and edema are the most common internal findings, although other conditions, such as signs of asphyxial death, brain edema, cerebral infarction, pulmonary edema, acute respiratory distress syndrome, myocardial infarction, and acute tubular necrosis have also been observed [42,43]. Autopsy findings are unremarkable in many cases [43].

Determining the cause of death in the case of overdose of new psychoactive materials can be challenging due to the lack of data about these substances. It also presents another difficulty: the emerging new substances are often not found during the toxicological screening (so a negative screening does not exclude the possibility of an overdose). The determination of the cause of death in these cases is always based on the detection and quantification of the substances from the cadaveric blood, evaluation of the toxicological results, autopsy and histological findings (including a thorough review of the scientific literature) and excluding all other diseases or factors possibly causing the death or contributing to it. The data from scientific literature were limited in regard to the two substances detected but based on the toxicological results and after excluding other possible causes of death, the cause of death in our reported case was determined as an overdose of SCRAs, namely MDMB-4en-PINACA and 4F-ABUTINACA. However, the exact mechanism is unclear: autopsy results (brain edema, internal congestion, petechial bleeding, pleural ecchymoses, and fluidity of the blood) suggest asphyxia and, hence, respiratory depression, yet the above-described circumstances suggest a sudden collapse and potential arrhythmia. Although MDMB-4en-PINACA was one of the most commonly used SCRA in recent years, only a small number of cases have been reported, and no fatal case clearly attributed exclusively to the use of MDMB-4en-PINACA has been described in the scientific literature, and there is no manuscript describing 4F-ABUTINACA use (fatal or non-fatal).

The reported case further emphasizes that the relationship between concentration and effects in the case of SCRA use is unpredictable, with mortality occurring at relatively low concentrations, even in young and healthy individuals.

## 7. Conclusions

MDMB-4en-PINACA and 4F-ABUTINCA are strong, potentially lethal SCRAs; the exact effects and outcome of their use are unpredictable. MDMB-4en-PINACA and 4F-ABUTINCA are potent, potentially lethal SCRAs; nevertheless, the precise effects and outcomes of their use are unknown.

## Figures and Tables

**Table 1:** Analytes of interest with ionization mode, retention time (R_t_), quantifier (*) and qualifier ion transitions and collision energies (CE).

Analyte	Ionization Mode	R_t_ (min)	Precursor Ion (*m*/*z*)	Product Ion (*m*/*z*)	CE (eV)
MDMB-4en-PINACA	ESI+	1.030	358.2	213.1 *	25
				145.0	40
				90.0	60
4F-ABUTINACA	ESI+	1.668	370.2	135.1 *	25
				107.1	45
				93.1	45

**Table 2:** Main autopsy and histological findings of the reported case.

Organ	Weight (g)	Macroscopical Finding	Histological Finding
Brain	1670	edema	edema
Heart	322	- ^1^	minimal subendocardial fibrosis
Lung	right: 497 left: 426	congestion, edema, pleural petechial bleeding, and ecchymoses	acute congestion, minimal interstitial edema, macrophages containing double refracting brown pigments
Liver	1750	congestion	acute congestion, mild steatosis
Kidneys	302	congestion	congestion
Suprarenal gland	N/A	- ^1^	congestion
Arteries	N/A	minimal atherosclerosis	- ^2^

N/A: not applicable; ^1^ no pathological change was observed; ^2^ not examined.

## Data Availability

All data are contained within the article.
